# Regional Patterns and Risk Factors of Hearing Loss: A Cross-Sectional Study Across Saudi Arabia

**DOI:** 10.7759/cureus.95324

**Published:** 2025-10-24

**Authors:** Abdullah D Alotaibi, Kamaleldin B Said, Ruba M Elsaid Ahmed, Bassam E Alshammari, Lama B Abdulkarim, Amal S Alotaibi, Bashayr N Alsuwayt, Razan S Alsubaie, Wafi A Alrashidi, Manar A Alghaslan, Anwar E Almallahi, Salem A Almejrad, Bader Alkharisi, Naif M Altamimi, Mona S Alonazi

**Affiliations:** 1 Department of Otolaryngology, College of Medicine, University of Ha'il, Ha'il, SAU; 2 Department of Pathology, College of Medicine, University of Ha'il, Ha'il, SAU; 3 Department of Dermatology, College of Medicine, University of Ha'il, Ha'il, SAU

**Keywords:** conductive hearing impairment, ent diseases, factors of hearing loss, hearing impairment, hearing loss, hearing-loss, regional influence in hl, sensory neural hearing loss (snhl), surveillance of ear problems

## Abstract

Introduction

Hearing loss (HL) is a major public health issue affecting communication, social interaction, and quality of life. Despite the global recognition of HL as a growing concern, there is a scarcity of region-specific data in Saudi Arabia. This study aims to understand the frequency and rates of HL in this region and the potential factors contributing to the disorder.

Methods

This descriptive cross-sectional study utilized a web-based, self-administered questionnaire created with Google Forms. Data were analyzed using IBM SPSS Statistics for Windows, Version 24 (Released 2016; IBM Corp., Armonk, New York, United States).

Results

In the 789 responses, 6.6% reported HL, with 62.4% from the Eastern region, of which 41.2% were employed and 31.7% were students. Despite 36.5% reporting HL for more than five years, only 21.2% sought treatment. Additionally, 19.2% reported diabetes, and 51.9% had a family history of HL. Ages 11-50 years showed female predominance (females, 59.1%; males, 41%). Age was significant in HL (p < 0.001) irrespective of gender (males, 6.8%; females, 6.4%; p = 0.838), reaching peaks at 17.2% in the 51-60-year group and 25% in the 71-80-year group, implying a potential risk (OR = 1.43; 95% CI: 0.78-2.81; p = 0.032). A total of 34.6% indicated genetics as the main cause, followed by noise (26.9%), while infections, stroke, and fever accounted for 19.2%. Air or sea travel (7.7%) was insignificant (p = 0.628), while head injuries (11.5%) and exposure to low or high altitudes were reported in 21.2%. Noise hazard (p = 0.048) and medication use (p = 0.017) were significant in HL.

Conclusion

Thus, we demonstrate that age, noise exposure, and inheritance history are major predisposing factors. However, the study was limited by a relatively small sample size. Future large-scale, cohort multicenter studies using factual clinical data would provide deeper insights into HL.

## Introduction

Hearing loss (HL) has been one of the major debilitating disorders leading to significant losses in the health and wealth of the population globally. According to estimates from the World Health Organization (WHO), approximately 400 million people have disabling HL, mostly as a result of cochlear abnormalities that may be age-related, noise-induced, or hereditary in origin. Despite the prevalence of diverse factors that influence HL, including genetics, climate, occupation, and age, there is a severe paucity of high-quality reports in the region. This study aims to assess the regional patterns and prevalence of HL across Saudi Arabia and to identify the major risk factors and underlying causes associated with the disorder.

HL is a common problem that impacts people of all ages. It is defined as the inability to hear the sounds within the normal hearing threshold of 20 dB, which can affect one ear or both ears [1]. Three types of deafness are categorized as follows: mixed, sensorineural, and conductive. A malfunction in the ear's sound-conducting system results in the development of conductive deafness. The lesion could be located anywhere from the external auditory canal to the stapes footplate [2]. An impairment in the cochlea, auditory nerve, neural pathway, or their central connections to the auditory cortex results in sensorineural deafness [3]. However, there are several factors that cause HL. For instance, it has been found that the relationship between HL and climate conditions is directly proportional and increases the progression of HL gradually. Combined exposure to environmental conditions such as noise and heat may enhance the risk of HL for employees in noisy settings by 1.39 times compared to those who only work in noisy environments [4]. Schmidt et al. suggest that weather changes affect the severity of symptoms in Ménière's disease (MD). In addition, it has been suggested that lower atmospheric pressure was related to a higher risk of an attack and significantly increased levels of vertigo, tinnitus, and aural completion in people enduring MD. Furthermore, high humidity additionally expanded the chances of experiencing an attack [4].

Global estimates of hearing losses vary widely depending on geographic locations and local genetic population structures, and environmental factors. Worldwide estimates showed that 16% of cases involving disabling HL in adults were attributed to occupational noise exposure [5-7]. Occupational noise-induced hearing loss (ONIHL) is characterized by partial or complete HL in one or both ears due to work-related factors. This typically develops gradually over several years [8] depending on influencing factor(s). Research suggests that in the United States, individuals in the construction, manufacturing, mining, agriculture, utility, transportation, military, and music industries face the highest risk of ONIHL [9-11]. In Saudi Arabia, the occurrence of hearing impairment among workers exposed to high noise levels varies between 15.8% and 57% [12-14]. Notably, older individuals with mild HL have twice the risk of developing dementia as a result. On the other hand, severe HL increases that risk fivefold [15] and develops into sequelae of other cognitive dysfunctions. The ONIHL is a complex yet preventable condition, which underscores the importance of understanding its mechanisms and affected populations for the development of effective preventive measures.

Reduced oxygen (hypoxia) and atmospheric pressure (hypobaria) are the primary characteristics of high altitude (HA), which is defined as an altitude higher than 2500 m above sea level [16,17]. Changes in air pressure during flight can cause eardrum pain, vertigo, HL, and eardrum perforation, among other consequences on the middle ear [18]. The type of aircraft, altitude, and passenger characteristics all affect the frequency of symptoms. According to a point prevalence survey, 10% of adults and 22% of children exhibited otoscopic evidence of injury to the eardrum, and 20% of adult and 40% of child passengers experienced negative pressure in the middle ear following their flight [19]. Data on the frequency of perforation, which appears to be incredibly uncommon in travelers on commercial aircraft, is limited. Furthermore, HL has been reported as a result of ear infections. This includes the herpes virus family's beta subfamily, such as the cytomegalovirus (CMV)[3]. Vertical transmission, sexual transmission, and contact transmission are all conceivable strategies of spreading CMV. Roughly 10-20% of children with HL are influenced by inherent CMV (cCMV). An infection that causes nongenetic SNH (sensorineural HL) and neurodevelopmental issues [20]. An important source of HSV-2 transmission is through sexual contact or exposure to the mother’s vaginal canal (during delivery), whereas HSV-1 is primarily transmitted through direct contact. In children with identified risk factors, routine hearing assessments should be conducted between two years and 30 months of age.. A positive HSV culture postpartum disease and an in-utero disease stay chance variables for lasting intrinsic, postponed, or dynamic HL in childhood. HSV contamination in newborn children can result in one-sided or two-sided HL, mild to severe SNHL, and genuine neurological complications. The SARS-CoV-2 infection, of the Coronavirus family, could lead to several malfunctions in the upper respiratory tract. The affiliation between SARS-CoV-2 and sudden sensorineural HL (SSNHL) has been detailed in several case instances; numerous SARS-CoV-2 varieties can result in otological symptoms [21]. Unfortunately, high-quality data on the prevalence of HL before and after the pandemic is limited. Since the whole Ha’il population was vaccinated, the study may also account for the effect of vaccines.

Depending on the gender apportionment at birth, there's a better frequency of intrinsic HL in full-term newborn children (1.8 per 1000 live births) in males than in females (1.2 per 1000 live births) among newborn children with hearing disabilities [22]. Male-dominated work environments, the nature of occupations [23], and undesirable behaviors like smoking have been connected to this. It is widely known that smokers are more prone to hearing issues than nonsmokers, especially when they smoke for extended periods [24]. On the other hand, there is a common notion that women are frequently more noise-resistant than men [25]. As such, there are striking gender incongruities in hearing patterns over the sound-related work, with premenopausal women regularly predominating compared to men of the same age. Besides, premenopausal women display inconstancy in their capacity to listen amid the menstrual cycle, with their capacity to listen being best at the top of estradiol or when the proportion of estradiol to progesterone falls. Women start to lose their capacity to listen around menopause; this loss is more discernible and happens more rapidly than in males of the same age [26]. Men may be more subject to HL, communicate better socially, and those who have a wider social status are regularly more prone to HL [27]. This may clarify the high rate of HL in men.

The affiliation between advanced age and the type of HL shows that patients within the more senior age groups were more likely to have sensorineural HL, while conductive HL was more common among the youth. The populace inhabiting a location for a long time was found to be more likely to have blended HL [28]. In any case, the most common cause of HL among Saudi Arabia's youth population is conductive HL, usually after ear diseases [29]; whereas sensorineural HL is more prevalent within the more seasoned population [30]. Similarly, reports indicate that the conductive HL types are more common in youngsters [31].

The worldwide prevalence rates of HL have been expanding relentlessly. A comprehensive analysis providing detailed data on HL from the Global Burden of Disease study between 1990 and 2019 was reported. Normal yearly rate changes (AAPCs) in HL and age-standardized predominance rate (ASPR), by sex, locale, and category, were calculated to measure the temporal predominance patterns. Within the previously mentioned ponder, worldwide rates of HL bounced from 7514.97 × 105 in 1990 to 14566.62×105 in 2019, whereas ASPR rose from 173.33×102 per 100,000 in 1990 to 177.56×102 per 100,000 in 2019. Time went through in hearing incapacity (a long time lived with incapacity YLD) increased from 220080.97 × 102 in 1990 to 402353.05 × 102 in 2019. The AAPC was 83.27 (95% CI 70.66, 95.88) × 10-3 in predominance and -72.87 (95% CI -92.18, -53.56) × 10-3 in YLD. Critical correlations of AAPCs with ASPR (r = -0.60, p < 0.001) and age-standardized YLD (r = -0.43, p = 0.0012 for YLD < 455, r = 0.32, p < 0.001 for YLD ≥ 455) were recognized. The YLDs of HL owing to word-related noise (HLOON) expanded from 39334.39 (95% UI 26881.04, 55999.67) × 102 in 1990 to 70014.49 (98% UI 47605.62, 100593.43) × 102 in 2019, and an increasing AAPC was observed among females aged 15 to 49 years globally and across most regions. The age effect was below zero in seven age groups, while the period effect for HL prevalence showed an increasing trend, and the birth cohort effect demonstrated a decreasing trend over time. The number of cases and ASPR of HL within the population is still growing. Efforts to control HL, especially HLOON, are imminent [32]. Despite the steadily rising incidence rates within the regions, including Saudi Arabia, there is a severe lack of high-quality data regarding the causes and contributing factors of HL across all age groups. Thus, such research is deemed necessary. This article was previously posted to the preprints server on September 23, 2024.

## Materials and methods

Study design

This study was a cross-sectional design using a web-based online public questionnaire sent out to different regions of Saudi Arabia, namely, Eastern, Western, Northern, and Southern. All messages were sent simultaneously within the same time frame. Data were collected from October 2023 to February 2024. A panel of reviewers, ENT specialists from the King Khalid Hospital and University of Ha’il clinics, reviewed the questionnaire and provided comments that were considered. Since over 97% of government utilities and services are now on digital electronic platforms, and most of the Saudi population is relatively young, he potential bias toward younger, more technologically inclined participants, and the corresponding exclusion of less tech-savvy groups, is unlikely to have a significant impact. We tested the questionnaire on a sample of participants from different age groups and found it to be user-friendly and suitable for all. 

Sample size and sampling procedure

The sample was a self-administered nonprobability sample of social media users in Saudi Arabia. The web-based data collection tool was designed using Google Forms and was distributed via social media applications, i.e., mainly WhatsApp, Twitter, and others. An invitation letter was sent via WhatsApp groups and was posted on some community groups on Twitter. The invitation letter explained the aim of the study and the approximate time required to complete the questionnaire. Participants were asked to distribute the survey further among their social networks. A total of 789 participants were screened from the responses.

Data collection tool

The questionnaire comprised the following demographic characteristics, including consent form, age, gender, health status and treatment history, region of residence, job type, if any, and other related factors prone to influence HL. The questionnaire was prepared in English and then translated into Arabic. Language validity was conducted by retranslating the Arabic version of the questionnaire into English to ensure that the original meaning of the questions was preserved (back-translation), which was done by the authors, who are influential native speakers in both English and Arabic.

Ethics approval

Ethics Approval was obtained from the Research Ethics Standing Committee, University of Hail, #UOH 2021-631, dated 4/3/2024, number H-2024-067. Informed consent was obtained from all participants before participation in this study. 

Statistical analysis

IBM SPSS Statistics for Windows, Version 24 (Released 2016; IBM Corp., Armonk, New York, United States) was used for the analysis of data collected. Descriptive and stratified analyses were conducted; we present absolute numbers, proportions, and graphical distributions. We conducted exact statistical tests for proportions and showed that p-values were appropriate (a p-value of <0.05 was considered statistically significant).

## Results

In this study, we provided the frequency of HL among different patient demographics across different regions in Saudi Arabia. In addition to patients’ demographics, family history, and genetics, climate, urbanization, and industrialization are the major differences between provinces that are prone to influencing HL. The study surveyed 789 participants and revealed that a small proportion (6.6%) reported being diagnosed with HL. Additionally, the sociodemographic analysis showed a diverse age distribution, with the majority falling between 11 and 50 years old and a slight female predominance (59.1%). The largest proportion of participants resided in the Eastern province (62.4%) and were either employed (41.2%) or students (31.7%) (Table 1).

**Table 1:** Sociodemographic characteristics (n = 789)

		N	%
Age (in years)	0-10	8	1.0
	11-20	147	18.6
	21-30	202	25.6
	31-40	173	21.9
	41-50	178	22.6
	51-60	64	8.1
	61-70	9	1.1
	71-80	4	.5
	81+	4	.5
Gender	Female	466	59.1
	Male	323	40.9
Residence	Northern province	198	25.1
	‏Eastern province	492	62.4
	Central province and Riyadh	79	10.0
	Southern province	2	.3
	Western province	18	2.3
Employment	Employee	325	41.2
	Student	250	31.7
	Unemployed	214	27.1
Diagnosed with hearing loss	No	737	93.4
	Yes	52	6.6

Individuals aged over 50 years had a higher prevalence of HL compared to younger age groups (p < 0.001). Specifically, among individuals aged 51-60 years, the prevalence of HL was 17.2%, which is notably higher than in the younger age categories. Moreover, the prevalence appears to increase with advancing age, with individuals aged 61-70 years showing a prevalence of 0.0% and those aged 71-80 years showing a prevalence of 25.0%. Among females, 30 out of 466 individuals were diagnosed with HL, accounting for 6.4% of the total female population. Similarly, among males, 22 out of 323 individuals were diagnosed with HL, accounting for 6.8% of the total male population. However, there was no significant difference in the prevalence of HL observed (p = 0.838) (Table 2).

**Table 2:** Prevalence of hearing loss based on age and gender A p-value of <0.05 was considered statistically significant

			Diagnosed with hearing loss		Total	p-value
			No	Yes		
Age (years)	0-10		6	2	8	<0.001
			75.0%	25.0%	100.0%	
	11-20		141	6	147	
			95.9%	4.1%	100.0%	
	21-30		196	6	202	
			97.0%	3.0%	100.0%	
	31-40		160	13	173	
			92.5%	7.5%	100.0%	
	41-50		167	11	178	
			93.8%	6.2%	100.0%	
	51-60		53	11	64	
			82.8%	17.2%	100.0%	
	61-70		9	0	9	
			100.0%	0.0%	100.0%	
	71-80		3	1	4	
			75.0%	25.0%	100.0%	
	81+		2	2	4	
			50.0%	50.0%	100.0%	
Gender	Female		436	30	466	0.838
			93.6%	6.4%	100.0%	
	Male		301	22	323	
			93.2%	6.8%	100.0%	

**Figure 1:**
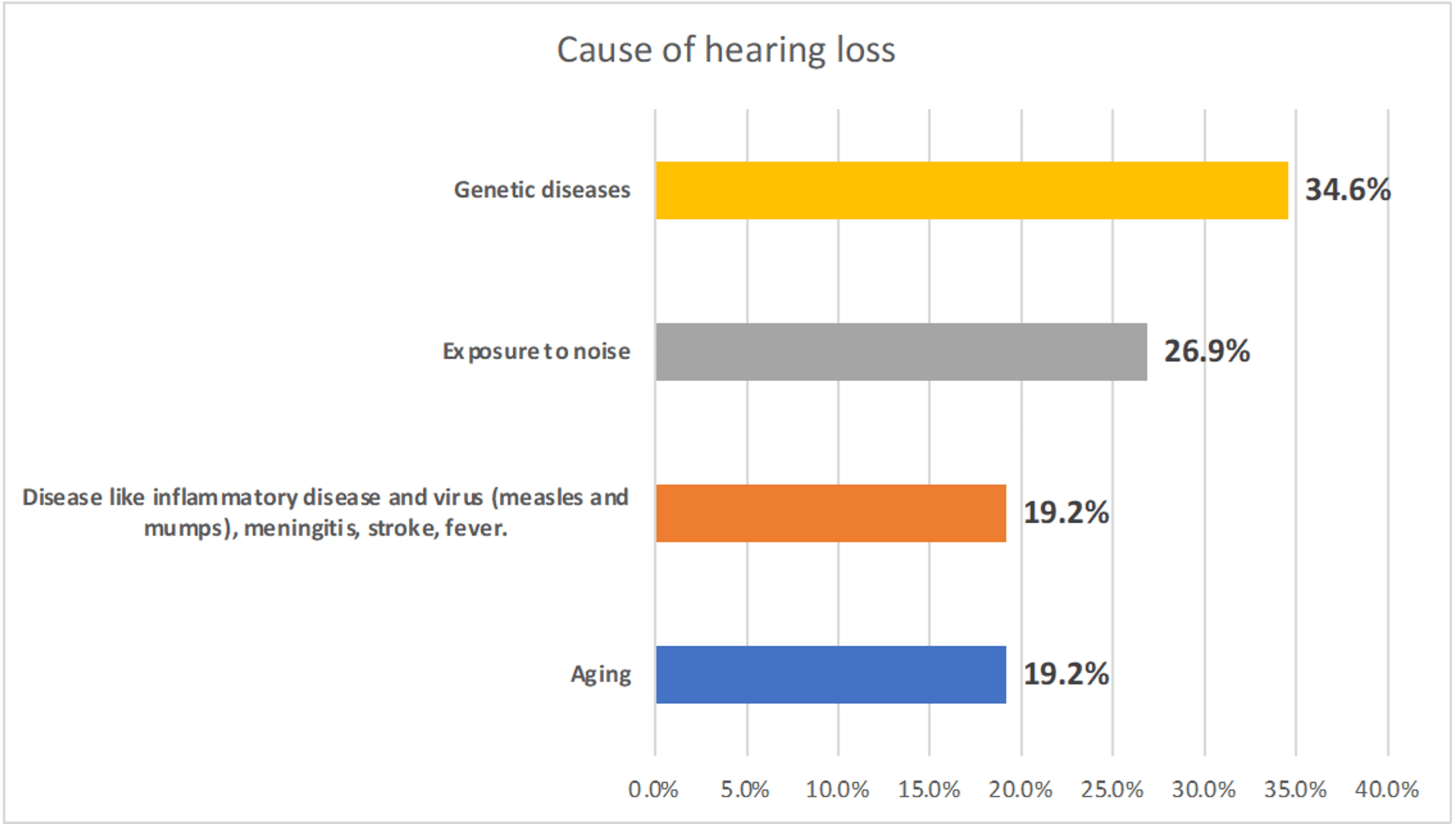
Potential causes of hearing loss among the respondents in the Ha’il region

Figure 1 illustrates the causes of HL among the surveyed individuals. Genetic diseases emerged as a potential cause, accounting for 34.6% of cases, followed closely by exposure to noise at 26.9%. Disease-related factors, including inflammatory conditions, viral infections such as measles and mumps, meningitis, stroke, and fever, as well as aging, each contributed to 19.2% of cases.

Other clinical characteristics related to HL are given in Table 3. Results indicate varied durations since diagnosis, with the largest proportion experiencing HL for more than five years (36.5%). A minority were programmed and fitted with hearing aids or cochlear implants (21.2%). Additionally, 19.2% of participants reported having diabetes mellitus, while 51.9% had a family history of HL and/or hearing problems. Few individuals reported work involving travel by air or sea (7.7%), a history of head injury or trauma (11.5%), or relocation between low- and high-altitude areas (21.2%). Approximately a quarter (25.0%) were currently taking medications, with aspirin being the most common (9.6%).

**Table 3:** Clinical characteristics related to hearing loss (n = 52). Data are presented as N (%)

		N	%
Time since hearing loss was diagnosed	Less than a year	11	21.2
	1-3 years	13	25.0
	3-5 years	9	17.3
	More than 5 years	19	36.5
Programmed and fitted with hearing aids or cochlear implants	No	41	78.8
	Yes	11	21.2
Have diabetes mellitus	No	42	80.8
	Yes	10	19.2
Family history of hearing loss and/or hearing problems	No	25	48.1
	Yes	27	51.9
Work involve traveling by air or sea	No	48	92.3
	Yes	4	7.7
History of head injury or trauma	No	46	88.5
	Yes	6	11.5
Moved from a low-altitude area to a high-altitude area or vice versa	No	41	78.8
	Yes	11	21.2
Currently taking any medications	No	39	75.0
	Yes	13	25.0
Medications used	Nothing	39	75.0
	Aspirin	5	9.6
	Carboplatin	1	1.9
	Diabetic medication	2	3.8
	Erythromycin	1	1.9
	Losartan potassium	1	1.9
	Statins	1	1.9
	Vitamins	1	1.9
	Others	1	1.9

Table 4 shows the relationship between travel history and HL within the surveyed population. The data show no significant association between frequent travel by plane (more than three times per year) and the diagnosis of HL (p = 0.628). Among those who did not frequently travel by plane, 93.2% had no HL, compared to 6.8% who did. Similarly, among frequent travelers, 94.3% had no HL, while 5.7% did.

**Table 4:** Relationship between travel history and hearing loss A p-value of <0.05 was considered statistically significant

			Diagnosed with hearing loss		Total	Test statistic	p-value
			No	Yes			
Travel by plane frequently ((more than 3 times per year)	No	N	604	44	648	χ² = 0.234 df=1	0.628
		%	93.2%	6.8%	100.0%		
	Yes	N	133	8	141		
		%	94.3%	5.7%	100.0%		

A logistic regression model was used to predict the risk of potential factors for HL among the participants (Table 5). Individuals aged over 50 years have a significantly elevated risk (OR = 1.43; 95% CI: 0.78-2.81; p = 0.032), while those engaged in certain occupations seem to have a reduced risk (OR = 0.32; 95% CI: 0.02-0.73; p = 0.862). Frequent exposure to noisy environments (OR = 1.54; 95% CI: 0.83-3.01; p = 0.048) and regular use of medications like aspirin or nonsteroidal anti-inflammatory drugs (NSAIDs) (OR = 2.15; 95% CI: 1.03-3.32; p = 0.017) are also associated with higher odds of HL. Additionally, individuals responded with a history of genetic disorder (p = 0.0432), although these would need experimental testing to confirm, and those with a history of inflammatory conditions (OR = 0.72; 95% CI: 0.32-1.87; p = 0.229) show notable associations, though the latter is not statistically significant. Other factors, such as gender, travel frequency by plane, diabetes, family history of HL, occupation involving navigation across the sky or sea, and a history of head injury, do not show statistically significant associations with hearing loss in this study.

**Table 5:** Predictive factors for hearing loss NSAIDs: nonsteroidal anti-inflammatory drugs A p-value of <0.05 was considered statistically significant. A logistic regression model was done to predict the risk of potential factor for hearing loss among the participants

	Odds ratio at 95% confidence interval	p-value
Gender	0.65 (0.21-1.42)	0.716
Age >50 years	1.43 (0.78-2.81)	0.032
Occupation	0.32 (0.02-0.73)	0.862
Travel by plane often (>3 times a year)	1.21 (0.57-2.52)	0.231
Diabetes	0.88 (0.36-1.92)	0.675
Family history of hearing loss	0.54 (0.02-1.34)	0.554
Suffer from a genetic disorder	2.23 (1.34-3.58)	0.432
Suffer from inflammatory	0.72 (0.32-1.87)	0.229
Frequent exposure to a noisy environment	1.54 (0.83-3.01)	0.048
Work involves navigating across the sky or the sea	0.77 (0.13-1.68)	0.868
Suffered an injury or blow to the head	1.04 (0.43-1.95)	0.714
Used medications such as Aspirin /NSAIDs frequently	2.15 (1.03-3.32)	0.017

## Discussion

In this study, we provided the rates of HL among different patient demographics across different regions in Saudi Arabia. In addition to patients’ demographics, family history, and genetics, climate, urbanization, and industrialization are the major differences between provinces that are prone to influencing HL. HL induces many negative effects in populations, including mental health and cognitive function. Based on epidemiologic evidence, an association exists between HL and increased risk of isolation, depression, increased risk of dementia, poorer balance, falls, hospitalizations, fatigue, and early mortality [33]. Saudi Arabia is one of the youngest populations globally; however, estimates of HL remain significant.

In this study, we found 7% as the overall HL among the examined population in this study. Although this is relatively small, it is an improvement from the 2003 survey (18.6%) in ages 10 to 20 [34]; however, this rate remains significant when considered in relation to the overall population and the impact this will have on lifestyle. Nevertheless, the ratio of a slight increase in HL among gender differences in this study (female, 59%; male, 41%) remained the same for decades since 2003 [34], and it is in fact related to the fact that female respondents were higher with that rate. This is further confirmed by the fact that the gender difference in the test diagnosed the ratio between females (6.4%) and males (6.8%) was not statistically significant (p = 0.838). This is consistent with earlier findings that there was no statistically significant association between gender and different attributes of HL among the Saudi population [30]. The Eastern provinces are characterized by an industrialized lifestyle in addition to significant climate change toward a more humid weather year-round; this justifies the increase in HL in this region (62.4%) in agreement with earlier studies [35]. Despite the significance, only 21% sought diagnosis and hearing aid or cochlear implants, and a quarter (25.0%) were currently taking medications, with aspirin being the most common (9.6%). This implied negligence and lack of awareness.

Consistent with widely published data, hearing impairment appears to increase incrementally in those over 50 years old and higher compared to younger age groups, with a significant value (p < 0.001) [36]. However, genetic disorders (34.6%) and family history (52%) were revealed as causes, but these findings would need to be substantiated and confirmed by genetic testing. These were followed closely by exposure to noise at 26.9%, while other factors such as inflammatory conditions, viral infections such as measles and mumps, meningitis, stroke, and fever, as well as aging, each contributed to 19.2% of cases. Genetic and congenital influences are commonly associated with developed countries, particularly with a uniform population genetic structure such as Japan [37,38]. However, while Saudi Arabia is fascinatingly homogeneous, genetic studies are limited that reveal experimentally confirmed data. Infection and inflammation are common causes of HL worldwide; an example is Malawi, where HIV clinical studies are intensively conducted, where 24% (n = 380) had HL due to AIDS [39]. It is widely known that diabetes plays a significant role in HL [40], and Saudi Arabia has a high rate of the disease [41], where 19.2% of participants in this study reported having diabetes mellitus. Interestingly, among the 52 patients with HL, 10 (19.2%) were diabetic. Subjects with type 2 diabetes show a higher rate of altered evoked otoacoustic emissions (e-OAEs) compared to healthy individuals. E-OAE dysfunction is associated neither with a lesion of the auditory nerve pathway nor with diabetic microvasculopathy [42]. Therefore, diabetes per se, regardless of microvascular and neuropathic complications, is related to potential HL. While travelling, injuries, trauma, and other factors were lower and may not have a high impact, 21.2% of respondents reported a change in altitude from low to high or vice versa.

To predict HL among respondents, a logistic regression analysis was conducted. This confirmed that age groups over 50 years showed a significantly higher risk (OR = 1.43; 95% CI: 0.78-2.81; p = 0.032). In addition, this test identified that exposure to noise (OR = 1.54; 95% CI: 0.83-3.01; p = 0.048) and medications like aspirin or NSAIDs (OR = 2.15; 95% CI: 1.03-3.32; p = 0.017) were also associated with elevated odds of HL. Furthermore, potential genetic disorder(p = 0.432) and family history of inflammation and infections (p = 0.229) showed slight associations. However, an important finding was that other factors, such as gender, travel frequency by plane, diabetes, family history of HL, occupation involving navigation across the sky or sea, and a history of head injury, did not strongly indicate statistically significant associations with HL in this study.

The study was limited by the use of a surveillance questionnaire, which lacked objective clinical data necessary to determine the factual factors and accurately diagnose the degree and type of HL in the country. However, as a baseline study, important trends in public opinion were revealed that need to be substantiated by experiments and objective data. In addition, since this was a short-term study, the sample size may not represent the breadth of the regional and national trends in HL within the population.

## Conclusions

We have examined hearing profiles and patterns of 789 responses from across four different provinces for HL where 6.6 % had the impairment. The diverse sociodemographic surveillance included wide age distribution with the majority falling between 11 and 50 years old with a slight female predominance (59.1%). The association with advances in age irrespective of gender differences as well as the wide time lag since diagnosis have significant clinical and public health implications in geriatric healthcare and management. Furthermore, the high impact of chronic diseases such as diabetes and use of medications implied significance of early screening and specific diagnosis and treatment strategies. Importantly, family history of infections, genetic disorder, noisy environment, although require further confirmation, emerged as potential causes of HL. Thus, while other occupational health parameters can also affect, this study reveals frequences of HL and the potential predisposing factors as age-related, chronic diseases, noise-induced, or hereditary in origin. These findings have significant clinical implications in geriatric management strategies and population genetic structure, family, and public health issues. Future large cohort multicenter study would gain more insights, particularly in the humid and industrialized Eastern regions that showed increased incidences in this study.

## Appendices

**Table 6:** Questionnaire used for data collection

Age	0-10	11-20		21-30			31-40			41-50				51-60			61-70		71-80				+81
Gender	Male											Female											
Residence	The northern area		Eastern area			Riyadh and central region									Western area					Southern area			
Job	Student				Employee													Unemployed					
Do you travel by plane often (more than 3 times a year)?	Yes										No												
Have you been diagnosed with hearing loss? “If you answered yes, complete the rest of the questions”	Yes										No												
What is the cause of hearing loss?	Genetic cause		Excessive exposure to noise and loud sounds					Due to diseases such as viral infections (such as measles or mumps), meningitis, stroke, and high body temperature.														Due to aging	
When were you diagnosed with hearing loss?	Less than one year				One to three years								More than three to five years							More than five years			
Have your hearing aids or cochlear implants been programmed and fitted?	Yes										No												
Do you have diabetes?	Yes										No												
Are there any hearing diseases in the family history? (mother, father, grandfather, grandmother, uncle, etc.).	Yes										No												
Does the nature of your work involve moving across the sky or sea? (By plane or ship)	Yes										No												
Have you suffered an injury or blow to the head?	Yes										No												
Did you move from living in a low area to a high area or vice versa?	Yes										No												
Do you use any medications? “If you answered yes, complete the last question"	Yes										No												
What medication/medications are used? (If it is not among the medications, write it in the “other” box)	Aspirin		Diuretics						Gentamycin							Tobramycin					Erythromycin		
	Vancomycin		Quinine						Cisplatin							Carboplatin					Other: …..		
